# Association between indicators of systemic inflammation biomarkers during puberty with breast density and onset of menarche

**DOI:** 10.1186/s13058-020-01338-y

**Published:** 2020-10-01

**Authors:** Karin B. Michels, Kristen Keller, Ana Pereira, Claire E. Kim, José L. Santos, John Shepherd, Camila Corvalan, Alexandra M. Binder

**Affiliations:** 1 Department of Epidemiology, Fielding School of Public Health, University of California, 650 Charles Young Drive South, Room 71-264 CHS, Los Angeles, CA 90095 USA; 2 Institute for Prevention and Cancer Epidemiology, Faculty of Medicine and Medical Center, University of Freiburg, Freiburg im Breisgau, Germany; 3 Department of Biostatistics, Fielding School of Public Health, University of California, Los Angeles, CA USA; 4 Institute of Nutrition and Food Technology, University of Chile, Santiago, Chile; 5 Department of Nutrition, Diabetes and Metabolism, School of Medicine, Pontificia Universidad Católica de Chile, Santiago, Chile; 6 Population Sciences in the Pacific Program (Cancer Epidemiology), University of Hawaiʻi Cancer Center, University of Hawaiʻi, Honolulu, HI USA

**Keywords:** Systemic inflammation, Breast Cancer, Breast density, Menarche, Adiposity, Puberty, Interleukin-6, Tumor necrosis factor receptor 2, C-reactive protein

## Abstract

**Background:**

Systemic inflammation may play a role in shaping breast composition, one of the strongest risk factors for breast cancer. Pubertal development presents a critical window of breast tissue susceptibility to exogenous and endogenous factors, including pro-inflammatory markers. However, little is known about the role of systemic inflammation on adolescent breast composition and pubertal development among girls.

**Methods:**

We investigated associations between circulating levels of inflammatory markers (e.g., interleukin-6 (IL-6), tumor necrosis factor receptor 2 (TNFR2), and C-reactive protein (CRP)) at Tanner stages 2 and 4 and breast composition at Tanner stage 4 in a cohort of 397 adolescent girls in Santiago, Chile (Growth and Obesity Cohort Study, 2006–2018). Multivariable linear models were used to examine the association between breast composition and each inflammatory marker, stratifying by Tanner stage at inflammatory marker measurement. Accelerated failure time models were used to evaluate the association between inflammatory markers concentrations at each Tanner stage and time to menarche.

**Results:**

In age-adjusted linear regression models, a doubling of TNFR2 at Tanner 2 was associated with a 26% (95% CI 7–48%) increase in total breast volume at Tanner 4 and a 22% (95% CI 10–32%) decrease of fibroglandular volume at Tanner 4. In multivariable models further adjusted for body fatness and other covariates, these associations were attenuated to the null. The time to menarche was 3% (95% CI 1–5%) shorter among those in the highest quartile of IL-6 at Tanner 2 relative to those in the lowest quartile in fully adjusted models. Compared to those in the lowest quartile of CRP at Tanner 4, those in the highest quartile experienced 2% (95% CI 0–3%) longer time to menarche in multivariable models.

**Conclusions:**

Systemic inflammation during puberty was not associated with breast volume or breast density at the conclusion of breast development among pubertal girls after adjusting for body fatness; however, these circulating inflammation biomarkers, specifically CRP and IL-6, may affect the timing of menarche onset.

## Background

Chronic inflammation is increasingly suspected to play a critical role in breast cancer risk and progression [1]. The association between chronic inflammation and tumorigenesis may be driven by an impact of inflammation on estrogen synthesis, as well as general cell proliferation, insulin resistance, and insulin-like growth factor I [2–4]. Notably, these downstream impacts of chronic inflammation may shape breast composition, one of the strongest predictors of breast cancer risk [5].

Pro-inflammatory markers, such as interleukin-6 (IL-6), tumor necrosis factor α (TNFα), and C-reactive protein (CRP), elevate in response to infection, tissue damage, and in active stressed states such as obesity [6]. These persistent triggering factors may disrupt the balance between the expressions of pro- and anti-inflammatory markers and initiate sustained subclinical systemic inflammation. Inflammatory markers are secreted by both inflammatory cells, but also normal and malignant mammary cells [4]. Overexpression of these markers may induce continuous cellular proliferation, genomic instability, and cellular membrane damage. Further, they may suppress the antitumor immune response and increase estrogen levels in breast tissue, which all may directly promote the steps of neoplastic transformation through increased breast density [7].

Prior studies suggest that increased levels of certain pro-inflammatory markers may stimulate breast tumor growth and proliferation. IL-6 is a pro-inflammatory cytokine that has been associated with both breast cancer risk and progression [8]. Tumor necrosis factor (TNF) receptor 2 (TNFR2) has also been shown to have pro-inflammatory effects and to initiate immune modulation as well as tissue regeneration [9]. TNFR2 is one of the two soluble receptors of the cytokines TNF and lymphotoxin-α, and TNFR2 directly promotes the proliferation of tumor cells while activating immunosuppressive cells [10]. CRP, an acute-phase inflammatory marker, is produced in the liver along with IL-6 and TNFα. Epidemiologic studies have found that elevated levels of CRP were associated with cancer progression by providing a permissive environment for recurrent tumor growth (notably in gastrointestinal and kidney malignancies and a few in breast malignancies), inducing DNA damage, and promoting angiogenesis [11].

One of the mechanisms by which these pro-inflammatory markers may be related to cancer risk is through an influence on estrogen production. IL-6 has been identified as a stimulating factor for aromatase, the enzyme responsible for estrogen production in adipose tissue via conversion of androstenedione to estrogen [4]. Overexpression of these cytokines influences the growth and progression of malignant epithelial cells [12]. Similar to IL-6, increased circulating levels of TNFR2 may influence breast cancer risk through its effect on the estrogen pathway. TNF-α increases the production of aromatase, which may lead to greater breast density [13]. While CRP’s role in the pathogenesis of cancer remains elusive, it has been suggested to contribute to the growth of breast density by stimulating local estrogen production [14]. The Study of Women’s Health Across the Nation (SWAN) study, a longitudinal cohort study examining CRP and mammographic density, reported that high levels of CRP were associated with a slower decline in percent breast density with age [15].

The relation between pro-inflammatory markers and estrogen production may also have implications for pubertal development, another key predictor of breast cancer risk [16]. Pubertal breast development follows a coordinated surge in adrenal hormones preceding the reactivation of hypothalamic–pituitary–ovarian axis and production of estrogen from the ovaries [17]. Peak breast density is then achieved near the end of puberty [18]. There is growing evidence that this period of rapid mammary development creates a critical window of exposure susceptibility that can shape future breast cancer risk. Slower pubertal tempo (i.e., a longer window) has been associated with both increased breast density in young women and increased breast cancer risk, independent of age of menarche [16, 19]. We have previously reported that exposure to specific endocrine-disrupting chemicals during childhood and puberty is associated with an increase in adolescent breast density [20]. Although pro-inflammatory markers may also influence estrogen signaling, no study has evaluated the relation between pro-inflammatory marker levels during this window and pubertal breast development.

We therefore examined the association between circulating levels of inflammatory markers at Tanner stages 2 and 4 and breast composition at Tanner stage 4, as well as age at menarche, in a cohort of adolescent girls in Santiago, Chile. We hypothesize this study will provide greater insight into the influence of pro-inflammatory markers on breast density, which may have implications for future breast cancer risk.

## Methods

### Study population

The present study includes 397 girls participating in the Growth and Obesity Cohort Study (GOCS) in Santiago, Chile, for whom blood samples were collected at Tanner stages 2 and/or 4 and breast density was measured at Tanner stage 4. The GOCS was initiated in 2006 and included 1190 children (515 girls) aged between 2.6 and 4 years in the National Nursery Schools Council Program in Santiago and are representative of low to middle-income Chilean children. Participants were singletons born at term (37–42 weeks), with a birth weight between 2500 and 4500 g, and free from conditions that could affect growth such as food allergies and genetic and metabolic diseases. Anthropometric, growth, and maturation assessments were performed at the Institute of Nutrition and Food Technology (INTA) Health Clinic approximately every 6 to 12 months, and biological specimens were collected at defined time points over follow-up. More details on recruitment procedures and study design have been published previously [21]. The study protocol was approved by the Ethics Committee of INTA, University of Chile. Written informed consent was obtained from all parents or guardians of children prior to the start of data collection. Additional written informed consent was received from a parent or guardian when the data collection protocol was revised. The children gave assent when they turned 7 years of age.

### Tanner staging and assessment of breast tissue density

Breast tissue density measurements were collected at Tanner stage 4. Starting in 2010, breast development was assessed during clinical visits by visual inspection using Tanner’s rating scale (Tanner), and for Tanner 2, by palpation by a single female dietitian (trained by a pediatric endocrinologist with a kappa = 0.9) [22]. Breast composition was derived from a two-compartment model of adipose and fibro-glandular tissues using software developed by Shepherd and colleagues [23]. Breast fibroglandular volume (FGV; cm^3^), total breast volume (BV; cm^3^), and fibroglandular volume % (FGV% = FGV/BV × 100) were measured in the left and right breast once girls reached Tanner stage 4 by dual-energy X-ray absorptiometry (DXA) with a protocol designed to quantify breast composition [23]. Each breast was scanned using the Prodigy DXA system (GE Healthcare, Madison, WI, USA) software version 13.6., series 200674. The dosage of radiation exhibited by this assessment is extremely low and is lower than that received during a transcontinental flight, limiting any significant health risks associated with this X-ray method [24]. The protocol does not require breast compression and its validity and precision for measuring breast density in girls at different Tanner stages has been demonstrated previously [18]. A quality control phantom containing reference breast density materials was scanned throughout the study to assure a stable calibration. Values of the left and right breast were averaged for all analyses.

### Processing of blood samples

Inflammatory markers were measured via fasting blood samples (10 mL), which were collected at Tanner stages 2 and 4. Inflammatory markers were measured at Tanner 2 and 4 stages to evaluate consistency in the relationship between inflammatory markers across pubertal stages and adolescent breast composition. Samples were centrifuged, and serum and buffy coat separated. Serum samples were stored at − 80 °C until further processing.

### Assessment of inflammatory markers

#### Interleukin-6 (IL-6)

IL-6 was measured by Quantikine® Colorimetric Sandwich high-sensitive ELISA assay from R & D Systems, Minneapolis, MN (www.rndsystems.com). The assay employs the quantitative sandwich enzyme immunoassay technique. A monoclonal antibody specific for IL-6 has been pre-coated onto a microtitre plate. After the addition of samples, standards, controls, and conjugates to the wells, IL-6 is sandwiched between the immobilized antibody and the enzyme-linked antibody specific to IL-6. Upon the addition of a substrate, a color is generated that is proportional to the amount of IL-6 present in the sample. The assay has a sensitivity of 0.094 pg/mL, and the day-to-day variabilities of the assay at concentrations of 0.49, 2.78, and 5.65 pg/mL are 9.6, 7.2, and 6.5%, respectively.

#### TNF receptor 2 (TNFR2)

TNFR2 was measured by an ELISA assay from R & D Systems. The assay employs the quantitative sandwich enzyme immunoassay technique. A monoclonal antibody specific for TNFR2 has been pre-coated onto a microtitre plate. After the addition of samples, standards, controls, and conjugates to the wells, TNFR2 is sandwiched between the immobilized antibody and the enzyme-linked antibody specific to TNFR2. Upon the addition of a substrate, a color is generated that is proportional to the amount of TNFR2 present in the sample. The assay has a sensitivity of 0.6 pg/mL. The day-to-day variabilities of the assay at concentrations of 89.9, 197, and 444 pg/mL are 5.1, 3.5, and 3.6%, respectively.

#### High sensitivity C-reactive protein (hsCRP)

Serum levels of CRP were measured with a high-sensitive kit (ELISA, Kit, sensitivity: 0.12 mg/L, interassay coefficient variation: 6.3%; BIOMERICA, Inc.) [25].

### Covariates

Covariates included in our analyses were assessed at Tanner stages 2 and 4. Weight and height were measured every 6–12 months during follow-up using standardized techniques by trained personnel as previously described [26]; height for age *z*-scores was calculated based on the World Health Organization (WHO) 2007 growth references [27]. Body fat percentage was estimated at each visit using Tanita-BC-418 MA bioelectrical impedance measurements (Tanita-Corporation, Tokyo, Japan), at a measurement frequency of 50 kHz (accuracy 0.1 kg) [28]. Information on birthweight (grams) was obtained from birth records. Participant ethnicity was categorized based on whether their parent’s surnames were of Mapuche origin (e.g., no indigenous surnames vs. one or more indigenous surnames) [29]. Maternal education was considered an indicator of socio-economic status and categorized by whether the participant’s mother self-reported any post-secondary education. We adjusted our statistical models for birthweight, height, fat percentage, ethnicity, and maternal education because they have been previously been associated with inflammatory markers [30–32]. These variables have also been associated with pubertal timing and development [33–35].

### Statistical analysis

Multivariable linear models were used to examine the association between log-transformed breast composition and each inflammatory marker, stratifying by Tanner stage at inflammatory marker measurement. Associations between Tanner 2 inflammatory markers and breast composition were prospective, whereas Tanner 4 inflammatory markers and breast composition were measured concurrently. Associations with log-transformed inflammatory marker concentration were first estimated adjusting for age at inflammatory marker measurement (“age-adjusted model”). We then considered models further adjusting for fat percentage at biomarker measurement (“age and body fatness-adjusted model”) and fully adjusted models including age and fat percentage at biomarker measurement, as well as ethnicity (no indigenous surnames vs. one or more indigenous surnames), birth weight, age- and sex-specific height *Z*-score, and maternal education (“multivariable-adjusted model”). For each inflammatory marker, we reported the relative change in each breast composition measurement per doubling of inflammatory marker concentration and corresponding 95% confidence interval (CI). To accommodate more complicated dose-response relations, we alternatively modeled the association between log-transformed breast composition and indicator variables for quartiles of inflammatory measurement, adjusting for the same covariates. Quartile cut points were determined on the original scale in the study population, stratifying by Tanner stage at inflammatory marker measurement (i.e., quartile cut points are Tanner stage specific). Based on these models, we reported the relative change in each breast composition measurement for each quartile relative to the lowest quartile and corresponding 95% CI.

Accelerated failure time models were used to evaluate the association between serum inflammatory marker concentration and time to menarche, stratifying by Tanner stage at biomarker measurement. Assuming a Weibull distribution, time to menarche was calculated as the time from birth to self-reported age at menarche. For the analysis of Tanner 4 inflammatory markers, individuals were excluded if menarche occurred before biomarker measurement (*n* = 39 left-censored); no participants were left-censored in the analysis of Tanner 2 inflammatory markers. Follow-up time for right-censored individuals was age at last visit. Similar to our models for breast composition, we modeled the association with log-transformed biomarker measurement, adjusting for the same covariates. We report the relative time to menarche (time ratio) per doubling of inflammatory marker and 95% CI. We also modeled the association between quartiles of biomarker measurement and the time to menarche. We report the estimated relative time to menarche for each quartile of inflammatory marker measurement compared to the first quartile. Wald tests were used to evaluate the statistical significance of the associations with inflammatory marker concentration when modeled as a continuous measure. When modeled categorically, a likelihood ratio test was used to evaluate whether the inclusion of inflammatory marker level quartiles significantly (alpha-level = 0.05) improved model fit relative to the model without indicators for inflammatory biomarker quartiles. To evaluate the statistical trend across quartiles, the log-transformed median within each quartile was included as a continuous covariate in the models. To compare whether the characteristics of those lost to follow-up after Tanner 2, or those who only consented to a blood draw at one of the Tanner stages, impact the estimated associations, sensitivity analyses were conducted restricting to the subset of individuals for which inflammatory markers were measured at both time points. All analyses were conducted in R version 3.6.2.

## Results

### Description of study population

**Fig. 1:**
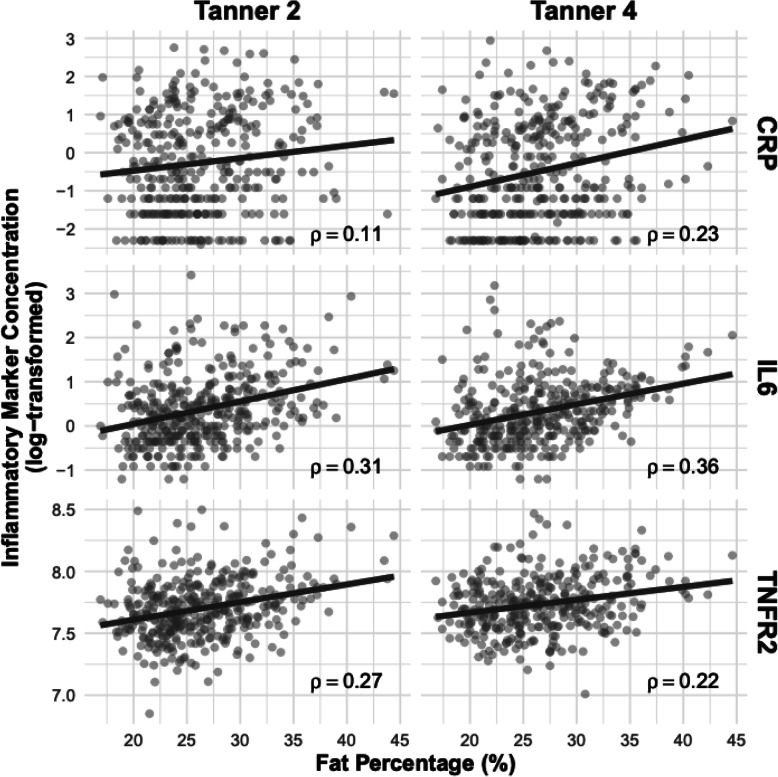
Spearman correlation between body fat percentage and log-transformed inflammatory markers at Tanner 2 and 4

Our study population at Tanner stage 2 included 397 girls with a mean age of 9.44 years, and 356 girls at Tanner 4 with a mean age of 11.16 years (Table 1). Of the 397 girls with inflammatory measurements at Tanner stage 2, 119 were lost to follow-up or did not consent to provide breast composition measurements at Tanner stage 4. These 119 participants tended to be older at the Tanner stage 2 visit, have a higher fat body fat percentage, and have higher levels of each inflammatory marker (Additional file 1). Very few participants provided a blood sample at Tanner stage 4 that did not consent to provide a breast composition measurement (*n* = 11). These 11 participants had slightly lower levels of the inflammatory markers, but otherwise had similar characteristics to those that provided breast composition measurements (Additional file 2). Their mean age at menarche was 11.8 years and 11.75 years, respectively. Plasma levels of CRP were measured in 238 girls at Tanner 2 and 271 at Tanner 4, with 237 having measurements at both time points. For IL-6 and TNFR2, plasma levels were measured in 266 girls at Tanner 2 and 269 at Tanner 4, with 263 having measurements at both time points. Plasma levels for IL-6 (2.10 vs. 1.93 pg/mL) and CRP (1.74 vs. 1.49 mg/L) were slightly higher in Tanner 2 than Tanner 4, while the opposite was found for TNFR2 (2270 vs. 2345 pg/mL) (Table 1). Correlations between the three inflammatory markers measured were low and marginally stronger in Tanner 4 compared to Tanner 2. Spearman correlations of the individual inflammatory markers at Tanner stage 2 vs. Tanner stage 4 were 0.31 and 0.36 for IL6, 0.27 and 0.22 for TNFR2, and 0.11 and 0.23 CRP, respectively. Levels of all three inflammatory markers were positively correlated with body fat percentage. Spearman correlations of individual inflammatory markers and body fat percentage were 0.31 and 0.36 for IL6, 0.27 and 0.22 for TNFR2, and 0.11 and 0.23 for CRP, at Tanner 2 and Tanner 4 respectively (Fig. 1). Fat percentage assessed by the Tanita scale was positively correlated with BV and inversely correlated with FGV; no correlation was observed with FGV%.

**Table 1:** Characteristics of pubertal girls at Tanner 2 and Tanner 4 in the Chilean Growth and Obesity Cohort Study

Characteristic	Tanner 2 (***n*** = 397)		Tanner 4 (***n*** = 356)	
	*N* missing	Distribution^A^	*N* missing	Distribution^A^
Age (years)	0	9.44 (1.31)	0	11.16 (0.84)
Age at menarche	52	11.80 (0.89)	24	11.75 (0.84)
Height (*Z*-score)	0	0.09 (0.96)	1	0.39 (1.00)
BMI (*Z*-score)	0	0.81 (1.12)	1	0.91 (1.05)
Fat percentage (%)	1	26.28 (4.88)	0	26.57 (5.06)
Maternal education	0		0	
No post-secondary education		304 (76.57)		268 (75.28)
Post-secondary education		93 (23.43)		88 (24.72)
Ethnicity	0		0	
No Mapuche background		327 (82.37)		294 (82.58)
Mapuche background		70 (17.63)		62 (17.42)
Birth weight (kg)	12	3.38 (0.39)	10	3.34 (0.39)
Birth length (cm)	12	49.77 (1.71)	10	49.68 (1.74)
Inflammatory biomarkers^B^				
C-reactive protein (CRP; mg/L)	44	1.74 (2.46)	2	1.49 (2.26)
Interleukin-6 (IL-6; pg/mL)	11	2.10 (2.70)	5	1.93 (2.29)
TNF receptor 2 (TNFR2; pg/mL)	11	2270.96 (579.53)	5	2345.29 (548.80)
Breast composition at Tanner 4				
Total breast volume (BV; cm^3^)	119	206.81 (95.65)	11	221.48 (100.79)
Fibroglandular volume (FGV; cm^3^)	119	81.50 (32.11)	11	82.59 (33.78)
Percent fibroglandular volume (%FGV; cm^3^)	119	43.38 (16.05)	11	41.36 (16.66)

^A^Mean (SD) for continuous measures; count (%) for categorical measures

^B^Range of inflammatory marker levels at Tanner 2: CRP 0.1–15.7 mg/L, IL-6 0.3–19.7 pg/mL, TNFR2 944.4–4902.5. Range of inflammatory markers at Tanner 4: CRP 0.1–18.9 mg/L, IL-6 0.3–24.0 pg/mL, TNFR2 1345.8–4548.1 pg/mL

### Associations with breast composition

**Fig. 2:**
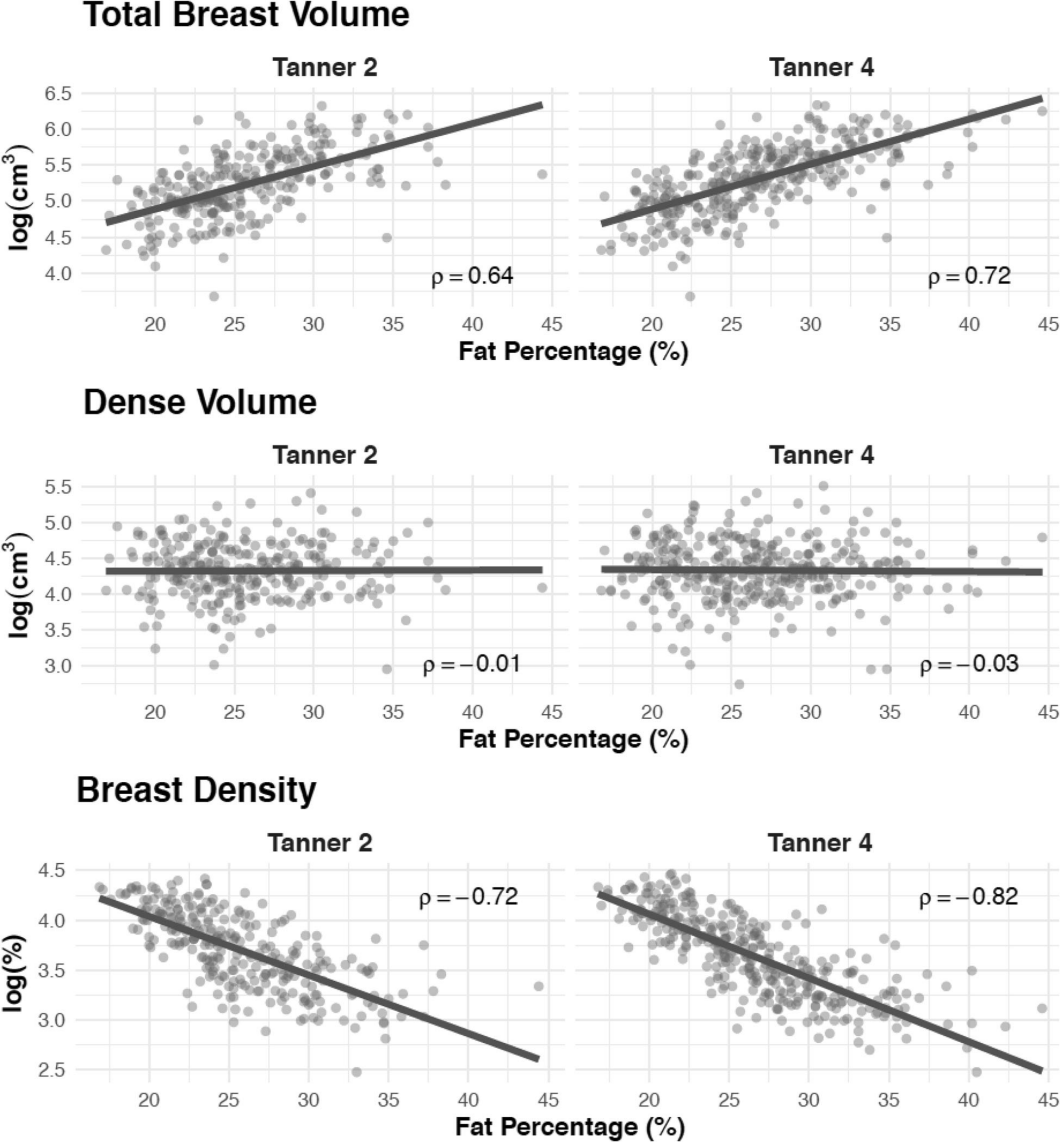
Spearman correlation between body fat percentage and breast composition at Tanner 2 and 4

In age-adjusted linear regression models, several statistically significant associations were found between inflammation marker levels measured at Tanner 2 and Tanner 4 and BV and FGV% (Table 2). For example, a doubling of TNFR2 at Tanner 2 was associated with a 26% (95% CI 7–48%) increase in breast volume at Tanner 4, and a 22% (95% CI 10–32%) decrease of FGV% at Tanner 4. However, after further adjustment for fat percentage estimated by bioimpedance, all associations were attenuated to the null (Table 2). Additional adjustment for ethnicity, birthweight, and age- and sex-specific *z*-score for height did not further change our estimated associations between inflammatory markers and breast composition (Table 2). These results indicate that body fat percentage was the most important confounder in both analyses: it had a strong positive correlation with BV at Tanner 2 and Tanner 4 (Spearman rho: 0.64 and 0.72, respectively) and a strong negative correlation with FGV%, at Tanner 2 and Tanner 4 (Spearman rho: − 0.72 and − 0.82, respectively; Fig. 2). When the inflammatory markers were categorized into quartiles, several statistically significant trends were observed between the inflammation marker serum levels and BV and density measure, but none of these associations prevailed after adjustment for body fatness (Table 3). Sensitivity analyses were performed restricting to the subset of individuals for which inflammatory markers were measured at both time points to appraise whether the characteristics of those lost to follow-up after Tanner 2, or those who only consented to a blood draw at one of the Tanner stages, impact the estimated associations. The associations with breast composition and inference among this subset of the study did not differ from our primary results (Additional files 3, 4).

**Table 2:** Association of inflammatory markers measured at Tanner 2 and Tanner 4 with breast composition measured at Tanner 4

Inflammatory marker	Breast Tanner stage	Relative change in breast composition per doubling of inflammatory marker					
		Age-adjusted model^A^		Age and body fatness-adjusted model^B^		Multivariable-adjusted model^C^	
		*N*	Estimate (95% CI)	*N*	Estimate (95% CI)	*N*	Estimate (95% CI)
Total breast volume							
CRP	Tanner 2	246	1.03 (1.00–1.06)	245	1.01 (0.99–1.04)	236	1.01 (0.99–1.04)
	Tanner 4	343	1.02 (1.00–1.05)	343	0.98 (0.97–1.00)	332	0.99 (0.97–1.01)
IL-6	Tanner 2	272	1.05 (1.00–1.10)*	271	0.98 (0.94–1.02)	262	0.98 (0.94–1.02)
	Tanner 4	341	1.10 (1.06–1.15)***	341	1.01 (0.97–1.04)	330	1.01 (0.97–1.04)
TNFR2	Tanner 2	272	1.26 (1.07–1.48)**	271	1.03 (0.90–1.18)	262	1.07 (0.93–1.22)
	Tanner 4	341	1.35 (1.15–1.57)***	341	1.07 (0.95–1.20)	330	1.06 (0.94–1.19)
Fibroglandular volume							
CRP	Tanner 2	246	1.00 (0.97–1.03)	245	1.00 (0.97–1.03)	236	1.00 (0.98–1.03)
	Tanner 4	343	0.98 (0.96–1.00)*	343	0.98 (0.96–1.00)*	332	0.98 (0.96–1.01)
IL-6	Tanner 2	272	0.99 (0.95–1.03)	271	0.99 (0.95–1.03)	262	0.99 (0.95–1.04)
	Tanner 4	341	1.00 (0.96–1.04)	341	1.00 (0.96–1.04)	330	1.01 (0.96–1.05)
TNFR2	Tanner 2	272	0.98 (0.85–1.13)	271	0.98 (0.85–1.14)	262	1.01 (0.87–1.17)
	Tanner 4	341	1.06 (0.92–1.21)	341	1.06 (0.92–1.23)	330	1.04 (0.91–1.20)
Percent fibroglandular volume							
CRP	Tanner 2	246	0.97 (0.95–0.99)*	245	0.99 (0.97–1.01)	236	0.99 (0.97–1.01)
	Tanner 4	343	0.96 (0.94–0.98)***	343	0.99 (0.98–1.01)	332	1.00 (0.98–1.01)
IL-6	Tanner 2	272	0.94 (0.90–0.98)**	271	1.01 (0.98–1.04)	262	1.01 (0.98–1.05)
	Tanner 4	341	0.90 (0.87–0.94)***	341	1.00 (0.97–1.02)	330	1.00 (0.97–1.02)
TNFR2	Tanner 2	272	0.78 (0.68–0.90)***	271	0.95 (0.86–1.05)	262	0.95 (0.85–1.05)
	Tanner 4	341	0.78 (0.68–0.90)***	341	1.00 (0.91–1.09)	330	0.98 (0.90–1.08)

^A^Linear regression model adjusting for age at inflammatory biomarker measurement

^B^Model adjusting for age at inflammatory biomarker measurement and fat percentage at biomarker measurement

^C^Model adjusting for age at inflammatory biomarker measurement, fat percentage at biomarker measurement, ethnicity, birth weight, height age- and sex-specific *Z*-score, and maternal education

*p < 0.05

**p < 0.01

***p < 0.001

**Table 3:** Association of quartiles of inflammatory markers measured at Tanner 2 and Tanner 4 with breast composition measured at Tanner 4

Inflammatory marker	Breast Tanner stage			Relative change in breast composition compared to Q1 (95% CI)^F^			
		*N*	LRT *p* value^D^	Q2	Q3	Q4	*p* for Trend^E^
**Age-adjusted model**^**A**^							
Total breast volume							
CRP	Tanner 2	246	0.054	1.20 (1.03–1.41)*	1.14 (0.98–1.32)	1.19 (1.02–1.38)*	0.037
	Tanner 4	343	0.199	1.15 (0.99–1.33)	1.10 (0.97–1.26)	1.11 (0.98–1.26)	0.131
IL-6	Tanner 2	272	0.036	1.13 (0.98–1.31)	1.19 (1.03–1.37)*	1.22 (1.05–1.42)*	0.010
	Tanner 4	341	< 0.0001	1.23 (1.08–1.40)**	1.27 (1.11–1.45)***	1.41 (1.24–1.61)***	< 0.0001
TNFR2	Tanner 2	272	0.001	1.02 (0.88–1.18)	1.23 (1.06–1.42)**	1.26 (1.09–1.47)**	< 0.001
	Tanner 4	341	0.002	1.18 (1.03–1.35)*	1.24 (1.08–1.42)**	1.29 (1.12–1.48)***	< 0.001
Fibroglandular volume							
CRP	Tanner 2	246	0.759	1.07 (0.94–1.24)	1.04 (0.91–1.19)	1.01 (0.89–1.16)	0.840
	Tanner 4	343	0.161	0.99 (0.87–1.13)	0.99 (0.88–1.11)	0.89 (0.80–0.99)*	0.066
IL-6	Tanner 2	272	0.687	0.98 (0.86–1.12)	0.96 (0.85–1.09)	1.05 (0.92–1.20)	0.536
	Tanner 4	341	0.156	1.03 (0.91–1.16)	0.89 (0.79–1.01)	0.99 (0.88–1.11)	0.432
TNFR2	Tanner 2	272	0.466	0.98 (0.86–1.11)	1.08 (0.95–1.23)	0.98 (0.86–1.12)	0.883
	Tanner 4	341	0.585	1.06 (0.94–1.20)	1.09 (0.96–1.23)	1.06 (0.93–1.20)	0.380
Percent fibroglandular volume							
CRP	Tanner 2	246	0.082	0.89 (0.78–1.02)	0.92 (0.81–1.04)	0.86 (0.76–0.97)*	0.023
	Tanner 4	343	0.001	0.87 (0.76–0.99)*	0.90 (0.80–1.01)	0.80 (0.72–0.90)***	< 0.001
IL-6	Tanner 2	272	0.005	0.86 (0.76–0.98)*	0.81 (0.72–0.92)***	0.86 (0.76–0.98)*	0.018
	Tanner 4	341	< 0.0001	0.84 (0.75–0.94)**	0.71 (0.63–0.79)***	0.70 (0.63–0.78)***	< 0.0001
TNFR2	Tanner 2	272	< 0.001	0.96 (0.85–1.09)	0.88 (0.77–0.99)*	0.78 (0.69–0.89)***	< 0.0001
	Tanner 4	341	0.017	0.91 (0.80–1.02)	0.88 (0.78–0.99)*	0.82 (0.72–0.93)**	0.002
**Age and body fatness-adjusted model**^**B**^							
Total breast volume							
CRP	Tanner 2	245	0.242	1.13 (1.00–1.29)	1.09 (0.96–1.23)	1.07 (0.95–1.22)	0.272
	Tanner 4	343	0.043	1.02 (0.92–1.14)	1.02 (0.93–1.12)	0.90 (0.82–0.99)*	0.077
IL-6	Tanner 2	271	0.986	1.01 (0.90–1.14)	1.02 (0.90–1.15)	1.00 (0.88–1.14)	0.991
	Tanner 4	341	0.191	1.09 (0.99–1.20)	0.98 (0.88–1.09)	1.04 (0.93–1.15)	0.906
TNFR2	Tanner 2	271	0.344	1.00 (0.89–1.12)	1.10 (0.97–1.24)	1.06 (0.93–1.20)	0.211
	Tanner 4	341	0.030	1.14 (1.03–1.25)*	1.14 (1.03–1.26)*	1.06 (0.96–1.18)	0.297
Fibroglandular volume							
CRP	Tanner 2	245	0.775	1.07 (0.93–1.24)	1.04 (0.91–1.19)	1.01 (0.88–1.16)	0.884
	Tanner 4	343	0.146	0.99 (0.87–1.12)	0.99 (0.88–1.11)	0.88 (0.79–0.99)*	0.060
IL-6	Tanner 2	271	0.694	0.98 (0.86–1.12)	0.96 (0.84–1.10)	1.05 (0.91–1.21)	0.540
	Tanner 4	341	0.161	1.02 (0.91–1.16)	0.89 (0.78–1.01)	0.98 (0.87–1.12)	0.465
TNFR2	Tanner 2	271	0.478	0.98 (0.86–1.11)	1.08 (0.94–1.23)	0.98 (0.85–1.13)	0.915
	Tanner 4	341	0.563	1.07 (0.94–1.20)	1.09 (0.96–1.24)	1.06 (0.94–1.21)	0.336
Percent fibroglandular volume							
CRP	Tanner 2	245	0.535	0.94 (0.86–1.04)	0.95 (0.87–1.05)	0.94 (0.86–1.04)	0.227
	Tanner 4	343	0.750	0.97 (0.89–1.05)	0.96 (0.90–1.04)	0.98 (0.91–1.05)	0.448
IL-6	Tanner 2	271	0.169	0.97 (0.88–1.06)	0.94 (0.86–1.04)	1.05 (0.95–1.16)	0.347
	Tanner 4	341	0.126	0.94 (0.87–1.01)	0.91 (0.84–0.98)*	0.95 (0.88–1.02)	0.169
TNFR2	Tanner 2	271	0.487	0.99 (0.90–1.08)	0.98 (0.89–1.08)	0.93 (0.84–1.02)	0.151
	Tanner 4	341	0.260	0.94 (0.87–1.01)	0.96 (0.89–1.03)	1.00 (0.92–1.08)	0.874
**Multivariable-adjusted model**^**C**^							
Total breast volume							
CRP	Tanner 2	236	0.314	1.12 (0.98–1.28)	1.08 (0.96–1.23)	1.08 (0.95–1.22)	0.234
	Tanner 4	332	0.247	1.03 (0.93–1.14)	1.02 (0.93–1.12)	0.94 (0.85–1.03)	0.286
IL-6	Tanner 2	262	0.951	1.01 (0.90–1.14)	1.03 (0.91–1.16)	1.00 (0.88–1.13)	0.983
	Tanner 4	330	0.096	1.11 (1.01–1.23)*	0.99 (0.89–1.10)	1.05 (0.95–1.17)	0.713
TNFR2	Tanner 2	262	0.132	0.99 (0.88–1.12)	1.13 (1.00–1.27)	1.08 (0.95–1.22)	0.097
	Tanner 4	330	0.036	1.12 (1.01–1.23)*	1.14 (1.03–1.27)*	1.04 (0.94–1.16)	0.468
Fibroglandular volume							
CRP	Tanner 2	236	0.787	1.07 (0.93–1.23)	1.05 (0.92–1.20)	1.03 (0.90–1.18)	0.695
	Tanner 4	332	0.556	0.99 (0.88–1.13)	0.99 (0.88–1.11)	0.92 (0.82–1.04)	0.236
IL-6	Tanner 2	262	0.671	0.99 (0.86–1.13)	0.97 (0.85–1.11)	1.06 (0.92–1.22)	0.429
	Tanner 4	330	0.139	1.05 (0.93–1.18)	0.90 (0.79–1.03)	1.01 (0.89–1.14)	0.650
TNFR2	Tanner 2	262	0.347	0.99 (0.87–1.13)	1.11 (0.97–1.27)	1.00 (0.87–1.15)	0.617
	Tanner 4	330	0.599	1.04 (0.92–1.18)	1.09 (0.96–1.24)	1.03 (0.91–1.17)	0.600
Percent fibroglandular volume							
CRP	Tanner 2	236	0.721	0.96 (0.87–1.06)	0.97 (0.88–1.07)	0.95 (0.87–1.05)	0.340
	Tanner 4	332	0.769	0.97 (0.89–1.05)	0.96 (0.90–1.04)	0.98 (0.91–1.06)	0.579
IL-6	Tanner 2	262	0.105	0.97 (0.88–1.07)	0.94 (0.86–1.04)	1.07 (0.96–1.18)	0.236
	Tanner 4	330	0.134	0.94 (0.87–1.02)	0.91 (0.84–0.99)*	0.95 (0.88–1.03)	0.226
TNFR2	Tanner 2	262	0.486	1.00 (0.91–1.10)	0.98 (0.89–1.08)	0.93 (0.84–1.03)	0.166
	Tanner 4	330	0.296	0.93 (0.86–1.01)	0.95 (0.88–1.03)	0.98 (0.91–1.07)	0.893

^A^Linear regression model adjusting for age at inflammatory biomarker measurement

^B^Model adjusting for age at inflammatory biomarker measurement and fat percentage at biomarker measurement

^C^Model adjusting for age at inflammatory biomarker measurement, fat percentage at biomarker measurement, ethnicity, birth weight, height age- and sex-specific *Z*-score, and maternal education

^D^Likelihood ratio test (LRT) *p* value for whether the addition of inflammatory biomarker quartiles improved model fit relative to the model without indicators for inflammatory biomarker quartiles

^E^Wald test *p* value for log-transformed median within each quartile included as a continuous covariate in models adjusting for age at inflammatory biomarker measurement, fat percentage at biomarker measurement, ethnicity, birth weight, height age- and sex-specific *Z*-score, and maternal education

^F^Inflammatory marker quartiles at Tanner 2: CRP (mg/L) [0.1–0.3], [0.4–0.7], [0.8–2.2], [2.3–15.7]; IL-6 (pg/mL) [0.3–0.8], [0.9–1.3], [1.4–2.2], [2.3–30.4]; TNFR2 (pg/mL) [944.4–1894.1], [1897.2–2169.0], [2171.9–2533.7], [2545.9–4902.5]. Inflammatory marker quartiles at Tanner 4: CRP (mg/L) [0.1–0.2], [0.2–0.5], [0.5–1.9], [2.0–18.9]; IL-6 (pg/mL) [0.3–0.8], [0.9–1.3], [1.4–2.1], [2.2–24.0]; TNFR2 (pg/mL) [1106.7–1960.4], [1971.3–2271.1], [2275.8–2638.1], [2639.9–4753.3]

*p < 0.05

**p < 0.01

***p < 0.001

### Associations with age at menarche

We also examined the association between inflammatory marker levels and the relative time to menarche from birth. A doubling of IL-6 levels at Tanner 2 was associated with a 1% (95% CI 0–1%) shorter time to menarche adjusting for age at biomarker measurement, which prevailed after adjustment for body fatness and other covariates (Table 4). This paralleled a 3% (95% CI 1–5%) shorter time to menarche among girls in the highest quartile of IL-6 at Tanner 2 relative to girls in the lowest quartile (Table 5), corresponding to an approximately 3.8 months earlier median age of menarche, based on our multivariable model. Given our models estimated the change in the relative time to menarche, this change on the absolute scale only holds for the difference in the median age of menarche between the highest and lowest quartiles of Il-6 at Tanner 2 and will differ for any other time point. We present this difference in months as an example of the implications at one specific time point. While Tanner 2 TNFR2 levels were also associated with time to menarche (Table 4), these associations did not persist when TNFR2 was modeled categorically (Table 5). A doubling of CRP at Tanner 4 was associated with a 1% (95% CI 0–1%) longer relative time to menarche, which also persisted after adjustment for covariates. These associations also manifested as significant trends when quartiles of inflammatory markers were modeled (Table 5). Notably, girls in the highest quartile of CRP at Tanner 4 had a 2% (95% CI 0–3%) longer time to menarche relative to the first quartile, corresponding to an approximately 2.6 month later median age of menarche in fully adjusted models. Associations restricting to those for which inflammatory markers were measured at both time points were consistent with the associations observed in the full study population (Additional files 5, 6).

**Table 4:** Association between inflammatory marker measurement at Tanner 2 and Tanner 4 with relative time to menarche

Inflammatory marker	Breast Tanner stage	*N*	Events^D^	Relative time to menarche per doubling of inflammatory marker; time ratio (95% CI)
**Age-adjusted model**^**A**^				
CRP	Tanner 2	348	302	1.00 (0.99–1.00)
	Tanner 4	298	291	1.01 (1.00–1.01)***
IL-6	Tanner 2	381	335	0.99 (0.99–1.00)*
	Tanner 4	298	291	1.00 (1.00–1.01)
TNFR2	Tanner 2	381	335	0.97 (0.95–0.99)*
	Tanner 4	298	291	1.00 (0.98–1.02)
**Age and body fatness-adjusted model**^**B**^				
CRP	Tanner 2	347	301	1.00 (0.99–1.00)
	Tanner 4	298	291	1.01 (1.00–1.01)***
IL-6	Tanner 2	380	334	0.99 (0.99–1.00)*
	Tanner 4	298	291	1.00 (0.99–1.01)
TNFR2	Tanner 2	380	334	0.97 (0.95–1.00)*
	Tanner 4	298	291	1.00 (0.98–1.01)
**Multivariable-adjusted model**^**C**^				
CRP	Tanner 2	336	291	1.00 (0.99–1.00)
	Tanner 4	291	284	1.01 (1.00–1.01)***
IL-6	Tanner 2	369	324	0.99 (0.98–1.00)**
	Tanner 4	291	284	1.00 (0.99–1.01)
TNFR2	Tanner 2	369	324	0.98 (0.96–1.00)*
	Tanner 4	291	284	1.00 (0.98–1.02)

^A^Accelerated failure time model for time to menarche from birth adjusting for age at inflammatory biomarker measurement

^B^Model adjusting for age at inflammatory biomarker measurement and fat percentage at biomarker measurement

^C^Model adjusting for age at inflammatory biomarker measurement, fat percentage at biomarker measurement, ethnicity, birth weight, height age- and sex-specific *Z*-score, and maternal education

^D^Number of girls reaching menarche during follow-up period

*p < 0.05

**p < 0.01

***p < 0.001

**Table 5:** Association of quartiles of inflammatory markers measured at Tanner 2 and Tanner 4 with relative time to menarche

Inflammatory marker	Breast Tanner stage				Relative time to menarche compared to Q1; time ratio (95% CI)^F^			
		*N*	Events^G^	LRT *p* value^D^	Q2	Q3	Q4	p for Trend^E^
**Age-adjusted model**^**A**^								
CRP	Tanner 2	348	302	0.167	0.99 (0.97–1.01)	1.00 (0.99–1.02)	0.98 (0.97–1.00)	0.299
	Tanner 4	298	291	< 0.0001	0.98 (0.97–1.00)*	1.01 (1.00–1.02)	1.02 (1.01–1.04)**	< 0.001
IL-6	Tanner 2	381	335	0.532	1.00 (0.98–1.02)	1.00 (0.98–1.02)	0.99 (0.97–1.01)	0.157
	Tanner 4	298	291	0.463	1.01 (0.99–1.02)	1.01 (1.00–1.03)	1.01 (1.00–1.03)	0.157
TNFR2	Tanner 2	381	335	0.103	1.01 (0.99–1.03)	1.00 (0.98–1.02)	0.98 (0.96–1.00)	0.047
	Tanner 4	298	291	0.217	1.01 (1.00–1.03)	1.01 (0.99–1.02)	1.00 (0.98–1.02)	0.666
**Age and body fatness-adjusted model**^**B**^								
CRP	Tanner 2	347	301	0.280	0.99 (0.97–1.01)	1.00 (0.99–1.02)	0.99 (0.97–1.01)	0.451
	Tanner 4	298	291	< 0.001	0.98 (0.97–1.00)*	1.01 (0.99–1.02)	1.02 (1.00–1.03)*	0.001
IL-6	Tanner 2	380	334	0.602	1.00 (0.98–1.02)	1.00 (0.98–1.02)	0.99 (0.97–1.01)	0.197
	Tanner 4	298	291	0.758	1.01 (0.99–1.02)	1.01 (0.99–1.02)	1.01 (0.99–1.02)	0.484
TNFR2	Tanner 2	380	334	0.121	1.01 (0.99–1.03)	1.00 (0.98–1.02)	0.98 (0.96–1.00)	0.058
	Tanner 4	298	291	0.136	1.01 (1.00–1.03)	1.00 (0.99–1.02)	1.00 (0.98–1.01)	0.365
**Multivariable-adjusted model**^**C**^								
CRP	Tanner 2	336	291	0.260	0.99 (0.97–1.01)	1.01 (0.99–1.02)	0.99 (0.97–1.01)	0.839
	Tanner 4	291	284	< 0.0001	0.98 (0.97–1.00)*	1.01 (1.00–1.02)	1.02 (1.00–1.03)*	< 0.001
IL-6	Tanner 2	369	324	0.048	1.00 (0.98–1.02)	1.00 (0.98–1.02)	0.97 (0.95–0.99)**	0.010
	Tanner 4	291	284	0.897	1.01 (0.99–1.02)	1.00 (0.99–1.02)	1.00 (0.99–1.02)	0.782
TNFR2	Tanner 2	369	324	0.062	1.01 (0.99–1.03)	0.99 (0.97–1.00)	0.98 (0.97–1.00)	0.044
	Tanner 4	291	284	0.128	1.02 (1.00–1.03)*	1.01 (0.99–1.02)	1.00 (0.99–1.02)	0.911

^A^Accelerated failure time model for time to menarche from birth adjusting for age at inflammatory biomarker measurement

^B^Model adjusting for age at inflammatory biomarker measurement and fat percentage at biomarker measurement

^C^Model adjusting for age at inflammatory biomarker measurement, fat percentage at biomarker measurement, ethnicity, birth weight, height age- and sex-specific *Z*-score, and maternal education

^D^Likelihood ratio test (LRT) *p* value for whether the addition of inflammatory biomarker quartiles improved model fit relative to the model without indicators for inflammatory biomarker quartiles

^E^Wald test *p* value for log-transformed median within each quartile included as a continuous covariate in models adjusting for age at inflammatory biomarker measurement, fat percentage at biomarker measurement, ethnicity, birth weight, height age- and sex-specific *Z*-score, and maternal education

^F^Inflammatory marker quartiles at Tanner 2: CRP (mg/L) [0.1–0.3], [0.4–0.7], [0.8–2.2], [2.3–15.7]; IL-6 (pg/mL) [0.3–0.8], [0.9–1.3], [1.4–2.2], [2.3–30.4]; TNFR2 (pg/mL) [944.4–1894.1], [1897.2–2169.0], [2171.9–2533.7], [2545.9–4902.5]. Inflammatory marker quartiles at Tanner 4: CRP (mg/L) [0.1–0.2], [0.2–0.5], [0.5–1.9], [2.0–18.9]; IL-6 (pg/mL) [0.3–0.8], [0.9–1.3], [1.4–2.1], [2.2–24.0]; THFR2 (pg/mL) [1106.7–1960.4], [1971.3–2271.1], [2275.8–2638.1], [2639.9–4753.3]

^G^Number of girls reaching menarche during follow-up period

* *p* < 0.05

**p < 0.01

***p < 0.001

## Discussion

To our knowledge, this is the first study of the association between circulating inflammatory markers and breast density among pubertal girls. Our results indicate that the association between inflammatory markers and adolescent breast density was driven by estimated body fat percentage. Regardless, our results are important to note as previous studies have found total BV to be associated with breast cancer risk among women with normal and lean body mass [36]. In contrast, the association between select inflammation markers and the onset of menarche persisted in our fully adjusted models.

Our results reinforce the relation between systematic inflammation and adiposity [37]. Growing epidemiologic evidence suggests inflammation may mediate the association between adiposity and disease development [37–39]. Adipose tissue secretes a range of hormones, cytokines, chemokines, and growth factors, which can alter insulin sensitivity, glucose metabolism, estrogen production, and inflammation [40, 41]. Several prior studies among adolescents have shown an association between body fatness and markers of chronic inflammation (e.g., IL-6, TNFα, and CRP) [42–45]. A recent study of adult female Chinese immigrants similarly found a positive association between CRP and TNFR2 and percent dense breast in unadjusted models [46]. For TNFR2, this association was similarly driven by non-dense breast area and was attenuated in adjusted models [46]. In contrast, CRP was associated with dense breast area, and the association with dense area persisted in fully adjusted models [46].

In our study, we observed disparate associations between specific inflammatory markers and age at menarche in our fully adjusted models. The robustness of these associations to adjustment for total body fat percentage suggests that inflammatory markers may be related to ovarian production of reproductive hormones. An increase in CRP levels at Tanner 4 was associated with longer relative time to menarche. Interestingly, a small (*n* = 25) study of Polish women reported that an earlier age at menarche was associated with higher levels of CRP in adulthood [47]. The inversion of this relation before and after menarche suggests that CRP may be associated with multiple facets of pubertal development. A cross-sectional study of US adolescents did not observe a significant association between CRP and self-reported pubertal status [48]. However, they did report a positive relation between pubertal status and IL-6 and an inverse relation with TNFα among both males and females [48]. In our longitudinal study, we did not observe an association between TNFR2 levels and age at menarche, but higher IL-6 levels at Tanner 2 were associated with an earlier age of menarche, which is consistent with the US study. Reproductive hormones, including estrogen and testosterone, increase substantially during puberty and are known to influence immune responses and inflammatory pathways [49]. We did not detect this same association in Tanner 4, which may be due to the loss of very early developers for the analysis of associations with Tanner 4 inflammatory markers (left-censored individuals). Our sensitivity analysis, assessing the degree to which selection bias may be contributing to differences in the estimated associations with biomarker levels at Tanner 2 and 4, indicated that that the association estimates and inference among individuals with biomarker measurements at both time points did not differ from our primary results. Overall, our results suggest that the associations between reproductive hormones and specific inflammatory markers are not consistent between Tanner 2 and Tanner 4 stages. Prior epidemiologic studies among postmenopausal women have reported differential effect of estrogen on CRP and IL-6, suggesting disparate mechanisms of action [50].

These inflammatory markers have previously been associated with breast cancer risk, particularly among postmenopausal women. The Health Aging and Body Composition study of women aged 70–79 years old evaluated baseline inflammatory markers (e.g., IL6, TNFRα, CRP) and incident breast cancer events. Due to the limited number of cases (*n* = 30–33), the authors were unable to reject the null hypothesis for IL6 and TNFRα (0.95 (95% CI 0.54–1.65); HR 0.70 (95% CI 0.29–1.72)) but observed a possible positive association with CRP (HR 1.32 (95% CI 0.91–1.93)) [6]. A meta-analysis similarly indicated that for each doubling of CRP concentration, risk of any breast cancer and postmenopausal breast cancer was elevated 7% (95% CI 2–12%) and 6% (95% CI 1–11%), respectively [51]. Another meta-analysis reviewing the association between obesity-related adipocytes and breast cancer among Asian women found that increased levels of TNFα was associated with increased risk of postmenopausal breast cancer. Specifically, the concentrations of IL6 and TNFα were higher in women with breast cancer with pooled mean difference of 2.15 (95% CI 1.64–2.66) and 1.70 (95% CI 1.10–2.30), respectively. The review also emphasized that higher levels of TNFα were more prevalent among women with BMI > 25 kg/m^2^ [52].

Strengths of our study include the prospective nature of the puberty cohort with repeated assessments of inflammatory markers, the unique availability of breast density assessments as a putative marker of future breast cancer risk, and the availability of well-measured correlated factors, such as body fatness. A limitation of our study is the number of participants that provided blood samples at Tanner 2 that did not consent to provide breast composition measurements at Tanner 4. This loss to follow-up may partially account for the disparate associations between our inflammatory markers measured at Tanner 2 and Tanner 4 and our outcomes of interest. In spite of this limitation, our study adds valuable new insight into the relation between inflammatory markers during puberty and breast development. The observed increase in systemic inflammation with increased body size may contribute to breast tissue damage during a critical period of development.

## Conclusions

In conclusion, indicators of systemic inflammation during puberty were not associated with adolescent breast volume or breast density after adjustment for body fatness; however, such circulating inflammation biomarkers may affect the timing of onset of menarche. While the exact mechanism by which inflammation plays a role in breast density development warrants further study, the shared positive relationship between total body fat, breast size, and pro-inflammatory markers may have important implications for future cancer risk.

## Supplementary information

**Additional file 1.** Study population characteristics of girls with measured inflammatory markers at Tanner stage 2 but no breast composition measurements at Tanner stage 4.

**Additional file 2.** Study population characteristics of girls with measured inflammatory markers at Tanner stage 4 but no breast composition measurements at Tanner stage 4.

**Additional file 3.** Association of inflammatory markers measured at Tanner 2 and Tanner 4 with breast composition measured at Tanner 4; restricting to individuals for which inflammatory markers were measured at Tanner 2 and Tanner 4.

**Additional file 4.** Association of quartiles of inflammatory markers measured at Tanner 2 and Tanner 4 with breast composition measured at Tanner 4; restricting to individuals for which inflammatory markers were measured at Tanner 2 and Tanner 4.

**Additional file 5.** Association of inflammatory marker measurement at Tanner 2 and Tanner 4 with relative time to menarche; restricting to individuals for which inflammatory markers were measured at Tanner 2 and Tanner 4.

**Additional file 6.** Association of quartiles of inflammatory markers measured at Tanner 2 and Tanner 4 with relative time to menarche; restricting to individuals for which inflammatory markers were measured at Tanner 2 and Tanner 4.

## Data Availability

The datasets used and/or analyzed during the current study are available from the corresponding author on reasonable request.

## Abbreviations

IL-6: Interleukin-6
TNFα: Tumor necrosis factor α
CRP: C-reactive protein
TNF: Tumor necrosis factor
TNFR2: Tumor necrosis factor receptor 2
SWAN: Study of Women’s Health Across the Nation
GOCS: Growth and Obesity Cohort Study
INTA: Institute of Nutrition and Food Technology
Tanner: Tanner’s rating scale
FGV: Breast fibroglandular volume
BV: Total breast volume
FGV%: Fibroglandular volume
DXA: Dual-energy X-ray absorptiometry
hsCRP: High sensitivity C-reactive protein
WHO: World Health Organization
CI: Confidence interval
