# Supplementary material for: Association between indicators of systemic inflammation biomarkers during puberty with breast density and onset of menarche
Source: Breast Cancer Res. 2020 Oct 1;22:104. doi: 10.1186/s13058-020-01338-y (PMC7531086; doi:10.1186/s13058-020-01338-y)
Supplement: Supplementary file 1 — Additional file 1. Study population characteristics of girls with measured inflammatory markers at Tanner stage 2 but no breast composition measurements at Tanner stage 4. [file 13058_2020_1338_MOESM1_ESM.docx]

**Additional File 1 Study population characteristics of girls with measured inflammatory markers at Tanner stage 2 but no breast composition measurements at Tanner stage 4**

|  | | **Breast Composition Measured at Tanner stage 4** | | | |
| --- | --- | --- | --- | --- | --- |
|  | | **Yes (n=278)** | | **No (n=** **119)** | |
| **Characteristic** | | N missing | Distribution* | N Missing | Distribution* |
| Age (years) | | 0 | 9.33 (1.29) | 0 | 9.70 (1.32) |
| Age at Menarche | | 23 | 11.79 (0.86) | 29 | 11.83 (0.98) |
| Height (Z-score) | | 0 | 0.14 (0.99) | 0 | -0.04 (0.90) |
| BMI (Z-score) | | 0 | 0.71 (1.04) | 0 | 1.03 (1.26) |
| Fat Percentage (%) | | 1 | 25.74 (4.52) | 0 | 27.52 (5.45) |
| Maternal Education | | 0 |  | 0 |  |
|  | No Post-Secondary Education |  | 212 (76.26) |  | 92 (77.31) |
|  | Post-Secondary Education |  | 66 (23.74) |  | 27 (22.69) |
| Ethnicity | | 0 |  | 0 |  |
|  | No Mapuche background |  | 228 (82.01) |  | 99 (83.19) |
|  | Mapuche background |  | 50 (17.99) |  | 20 (16.81) |
| Birth Weight (kg) | | 10 | 3.35 (0.39) | 2 | 3.46 (0.38) |
| Birth Length (cm) | | 10 | 49.71 (1.73) | 2 | 49.90 (1.67) |
| Inflammatory Biomarkers | |  |  |  |  |
|  | C-Reactive Protein (CRP; mg/L) | 32 | 1.71 (2.62) | 12 | 1.80 (2.05) |
|  | Interleukin-6 (IL-6; pg/mL) | 6 | 1.90 (2.17) | 5 | 2.57 (3.65) |
|  | TNF receptor 2 (TNFR2; pg/mL) | 6 | 2222.07 (551.59) | 5 | 2387.61 (628.61) |

*Mean (SD) for continuous measures; count (%) for categorical measures
