# Supplementary material for: Association between indicators of systemic inflammation biomarkers during puberty with breast density and onset of menarche
Source: Breast Cancer Res. 2020 Oct 1;22:104. doi: 10.1186/s13058-020-01338-y (PMC7531086; doi:10.1186/s13058-020-01338-y)
Supplement: Supplementary file 2 — Additional file 2. Study population characteristics of girls with measured inflammatory markers at Tanner stage 4 but no breast composition measurements at Tanner stage 4. [file 13058_2020_1338_MOESM2_ESM.docx]

**Additional File 2. Study population characteristics of girls with measured inflammatory markers at Tanner stage 4 but no breast composition measurements at Tanner stage 4**

|  | | **Breast Composition Measured at Tanner stage 4** | | | |
| --- | --- | --- | --- | --- | --- |
|  | | **Yes (n=345)** | | **No (n=** **11)** | |
| **Characteristic** | | N missing | Distribution* | N Missing | Distribution* |
| Age (years) | | 0 | 11.17 (0.84) | 0 | 10.67 (0.81) |
| Age at Menarche | | 23 | 11.77 (0.83) | 21 | 11.10 (0.81) |
| Height (Z-score) | | 1 | 0.38 (1.00) | 0 | 0.83 (1.01) |
| BMI (Z-score) | | 1 | 0.91 (1.05) | 0 | 0.97 (1.11) |
| Fat Percentage (%) | | 0 | 26.57 (5.10) | 0 | 26.77 (3.97) |
| Maternal Education | | 0 |  | 0 |  |
|  | No Post-Secondary Education |  | 260 (75.36) |  | 8 (72.73) |
|  | Post-Secondary Education |  | 85 (24.64) |  | 3 (27.27) |
| Ethnicity | | 0 |  | 0 |  |
|  | No Mapuche background |  | 284 (82.32) |  | 10 (90.91) |
|  | Mapuche background |  | 61 (17.68) |  | 1 (9.09) |
| Birth Weight (kg) | | 10 | 3.34 (0.39) | 0 | 3.54 (0.46) |
| Birth Length (cm) | | 10 | 49.69 (1.74) | 0 | 49.55 (1.63) |
| Inflammatory Biomarkers | |  |  |  |  |
|  | C-Reactive Protein (CRP; mg/L) | 2 | 1.51 (2.29) | 0 | 0.93 (0.95) |
|  | Interleukin-6 (IL-6; pg/mL) | 4 | 1.95 (2.31) | 1 | 1.43 (1.07) |
|  | TNF receptor 2 (TNFR2; pg/mL) | 4 | 2345.48 (548.39) | 1 | 2338.83 (593.19) |

*Mean (SD) for continuous measures; count (%) for categorical measures
