# Supplementary material for: Association between indicators of systemic inflammation biomarkers during puberty with breast density and onset of menarche
Source: Breast Cancer Res. 2020 Oct 1;22:104. doi: 10.1186/s13058-020-01338-y (PMC7531086; doi:10.1186/s13058-020-01338-y)
Supplement: Supplementary file 3 — Additional file 3. Association of inflammatory markers measured at Tanner 2 and Tanner 4 with breast composition measured at Tanner 4; restricting to individuals for which inflammatory markers were measured at Tanner 2 and Tanner 4. [file 13058_2020_1338_MOESM3_ESM.docx]

**Additional File 3. Association of inflammatory markers measured at Tanner 2 and Tanner 4 with breast composition measured at Tanner 4; restricting to individuals for which inflammatory markers were measured at Tanner 2 and Tanner 4**

| Inflammatory Marker | Breast Tanner Stage | Relative Change in Breast Composition Per Doubling of Inflammatory Marker | | | | | |
| --- | --- | --- | --- | --- | --- | --- | --- |
|  |  | Age-Adjusted Model^A^ | | Age and Body Fatness Adjusted Model^B^ | | Multivariable-Adjusted Model^C^ | |
|  |  | N | Estimate (95% CI) | N | Estimate (95% CI) | N | Estimate (95% CI) |
| Total Breast Volume | | | | | | | |
| CRP | Tanner 2 | 232 | 1.03 (1.00-1.06) | 231 | 1.01 (0.99-1.04) | 223 | 1.01 (0.99-1.04) |
|  | Tanner 4 | 263 | 1.01 (0.98-1.04) | 263 | 0.98 (0.96-1.00) | 253 | 0.99 (0.97-1.01) |
| IL-6 | Tanner 2 | 258 | 1.05 (1.00-1.11)* | 257 | 0.97 (0.93-1.02) | 249 | 0.97 (0.93-1.01) |
|  | Tanner 4 | 261 | 1.08 (1.02-1.14)** | 261 | 1.00 (0.96-1.04) | 251 | 1.00 (0.96-1.04) |
| TNFR2 | Tanner 2 | 258 | 1.25 (1.06-1.47)** | 257 | 1.03 (0.90-1.17) | 249 | 1.06 (0.93-1.21) |
|  | Tanner 4 | 261 | 1.33 (1.12-1.59)** | 261 | 1.09 (0.96-1.24) | 251 | 1.08 (0.95-1.23) |
| Fibroglandular Volume | | | | | | | |
| CRP | Tanner 2 | 232 | 1.00 (0.97-1.02) | 231 | 1.00 (0.97-1.02) | 223 | 1.00 (0.97-1.03) |
|  | Tanner 4 | 263 | 0.98 (0.96-1.01) | 263 | 0.98 (0.96-1.00) | 253 | 0.99 (0.96-1.01) |
| IL-6 | Tanner 2 | 258 | 0.98 (0.94-1.03) | 257 | 0.98 (0.93-1.03) | 249 | 0.98 (0.93-1.03) |
|  | Tanner 4 | 261 | 0.99 (0.94-1.03) | 261 | 0.98 (0.94-1.03) | 251 | 0.98 (0.94-1.03) |
| TNFR2 | Tanner 2 | 258 | 0.98 (0.85-1.12) | 257 | 0.97 (0.84-1.12) | 249 | 1.00 (0.86-1.15) |
|  | Tanner 4 | 261 | 1.06 (0.91-1.23) | 261 | 1.06 (0.90-1.23) | 251 | 1.04 (0.89-1.21) |
| Percent Fibroglandular Volume | | | | | | | |
| CRP | Tanner 2 | 232 | 0.97 (0.95-1.00)* | 231 | 0.99 (0.97-1.01) | 223 | 0.99 (0.97-1.01) |
|  | Tanner 4 | 263 | 0.97 (0.95-0.99)* | 263 | 1.00 (0.98-1.01) | 253 | 1.00 (0.98-1.01) |
| IL-6 | Tanner 2 | 258 | 0.93 (0.89-0.97)** | 257 | 1.01 (0.97-1.04) | 249 | 1.01 (0.97-1.04) |
|  | Tanner 4 | 261 | 0.91 (0.87-0.95)*** | 261 | 0.98 (0.96-1.01) | 251 | 0.98 (0.96-1.01) |
| TNFR2 | Tanner 2 | 258 | 0.78 (0.68-0.90)*** | 257 | 0.95 (0.85-1.05) | 249 | 0.94 (0.85-1.05) |
|  | Tanner 4 | 261 | 0.79 (0.68-0.92)** | 261 | 0.97 (0.88-1.06) | 251 | 0.96 (0.87-1.06) |

^A^Linear regression model adjusting for age at inflammatory biomarker measurement

^B^Model adjusting for age at inflammatory biomarker measurement and fat percentage at biomarker measurement

^C^Model adjusting for age at inflammatory biomarker measurement, fat percentage at biomarker measurement, ethnicity, birth weight, height age- and sex-specific Z-score, and maternal education

* p <0.05

** p <0.01

***p<0.001
