# Supplementary material for: Association between indicators of systemic inflammation biomarkers during puberty with breast density and onset of menarche
Source: Breast Cancer Res. 2020 Oct 1;22:104. doi: 10.1186/s13058-020-01338-y (PMC7531086; doi:10.1186/s13058-020-01338-y)
Supplement: Supplementary file 4 — Additional file 4. Association of quartiles of inflammatory markers measured at Tanner 2 and Tanner 4 with breast composition measured at Tanner 4; restricting to individuals for which inflammatory markers were measured at Tanner 2 and Tanner 4. [file 13058_2020_1338_MOESM4_ESM.docx]

**Additional File 4. Association of quartiles of inflammatory markers measured at Tanner 2 and Tanner 4 with breast composition measured at Tanner 4; restricting to individuals for which inflammatory markers were measured at Tanner 2 and Tanner 4**

|  | Inflammatory Marker | Breast Tanner Stage |  | | Inflammatory Marker Quartiles (ref: Q1)^F^ | | |  |
| --- | --- | --- | --- | --- | --- | --- | --- | --- |
|  |  |  | N | LRT^D^ | Q2 | Q3 | Q4 | Trend^E^ |
| **Age-Adjusted Model^A^** | | | | | | | | |
|  | Total Breast Volume | | | | | | | |
|  | CRP | Tanner 2 | 232 | 0.110 | 1.18 (1.01-1.39)* | 1.11 (0.95-1.30) | 1.18 (1.01-1.38)* | 0.066 |
|  |  | Tanner 4 | 263 | 0.738 | 1.09 (0.92-1.29) | 1.06 (0.91-1.22) | 1.06 (0.91-1.22) | 0.462 |
|  | IL-6 | Tanner 2 | 258 | 0.044 | 1.15 (0.99-1.34) | 1.18 (1.02-1.37)* | 1.22 (1.05-1.43)* | 0.013 |
|  |  | Tanner 4 | 261 | 0.003 | 1.19 (1.03-1.38)* | 1.26 (1.08-1.46)** | 1.29 (1.10-1.50)** | <0.001 |
|  | TNFR2 | Tanner 2 | 258 | 0.006 | 1.05 (0.90-1.21) | 1.22 (1.05-1.42)* | 1.26 (1.08-1.47)** | <0.001 |
|  |  | Tanner 4 | 261 | 0.028 | 1.19 (1.02-1.38)* | 1.17 (1.00-1.37) | 1.25 (1.07-1.46)** | 0.008 |
|  | Fibroglandular Volume | | | | | | | |
|  | CRP | Tanner 2 | 232 | 0.951 | 1.04 (0.90-1.20) | 1.02 (0.89-1.17) | 1.01 (0.88-1.16) | 0.936 |
|  |  | Tanner 4 | 263 | 0.456 | 0.99 (0.86-1.15) | 0.98 (0.87-1.11) | 0.91 (0.81-1.03) | 0.164 |
|  | IL-6 | Tanner 2 | 258 | 0.746 | 0.99 (0.87-1.13) | 0.95 (0.83-1.08) | 1.02 (0.89-1.17) | 0.906 |
|  |  | Tanner 4 | 261 | 0.506 | 1.01 (0.89-1.15) | 0.92 (0.81-1.05) | 0.97 (0.85-1.11) | 0.385 |
|  | TNFR2 | Tanner 2 | 258 | 0.817 | 1.01 (0.89-1.16) | 1.05 (0.92-1.20) | 0.99 (0.86-1.13) | 0.997 |
|  |  | Tanner 4 | 261 | 0.638 | 1.08 (0.95-1.22) | 1.08 (0.94-1.24) | 1.05 (0.92-1.20) | 0.516 |
|  | Percent Fibroglandular Volume | | | | | | | |
|  | CRP | Tanner 2 | 232 | 0.090 | 0.88 (0.77-1.01) | 0.92 (0.81-1.04) | 0.86 (0.75-0.98)* | 0.035 |
|  |  | Tanner 4 | 263 | 0.120 | 0.92 (0.80-1.06) | 0.93 (0.82-1.05) | 0.86 (0.76-0.97)* | 0.024 |
|  | IL-6 | Tanner 2 | 258 | 0.003 | 0.86 (0.76-0.98)* | 0.80 (0.71-0.91)*** | 0.84 (0.73-0.96)** | 0.006 |
|  |  | Tanner 4 | 261 | <0.0001 | 0.85 (0.75-0.96)** | 0.73 (0.64-0.83)*** | 0.75 (0.66-0.86)*** | <0.0001 |
|  | TNFR2 | Tanner 2 | 258 | <0.001 | 0.97 (0.86-1.10) | 0.87 (0.76-0.99)* | 0.79 (0.69-0.89)*** | <0.0001 |
|  |  | Tanner 4 | 261 | 0.077 | 0.91 (0.80-1.04) | 0.93 (0.81-1.06) | 0.84 (0.73-0.96)** | 0.015 |
| **Age and Body Fatness Adjusted Model^B^** | | | | | | | | |
|  | Total Breast Volume | | | | | | | |
|  | CRP | Tanner 2 | 231 | 0.406 | 1.12 (0.98-1.28) | 1.07 (0.95-1.22) | 1.07 (0.94-1.21) | 0.346 |
|  |  | Tanner 4 | 263 | 0.131 | 1.02 (0.91-1.15) | 1.01 (0.92-1.12) | 0.90 (0.82-1.00) | 0.119 |
|  | IL-6 | Tanner 2 | 257 | 0.890 | 1.03 (0.91-1.16) | 1.01 (0.89-1.14) | 0.97 (0.85-1.11) | 0.628 |
|  |  | Tanner 4 | 261 | 0.576 | 1.06 (0.95-1.19) | 0.99 (0.88-1.11) | 1.02 (0.91-1.15) | 0.963 |
|  | TNFR2 | Tanner 2 | 257 | 0.613 | 1.02 (0.90-1.15) | 1.08 (0.95-1.23) | 1.06 (0.93-1.21) | 0.269 |
|  |  | Tanner 4 | 261 | 0.054 | 1.15 (1.03-1.28)* | 1.13 (1.01-1.27)* | 1.06 (0.95-1.19) | 0.368 |
|  | Fibroglandular Volume | | | | | | | |
|  | CRP | Tanner 2 | 231 | 0.959 | 1.04 (0.90-1.20) | 1.02 (0.89-1.16) | 1.00 (0.87-1.15) | 0.992 |
|  |  | Tanner 4 | 263 | 0.380 | 0.99 (0.86-1.14) | 0.98 (0.87-1.11) | 0.90 (0.80-1.02) | 0.130 |
|  | IL-6 | Tanner 2 | 257 | 0.746 | 0.99 (0.87-1.13) | 0.94 (0.82-1.08) | 1.01 (0.88-1.17) | 0.959 |
|  |  | Tanner 4 | 261 | 0.450 | 1.01 (0.88-1.14) | 0.91 (0.79-1.04) | 0.96 (0.84-1.10) | 0.333 |
|  | TNFR2 | Tanner 2 | 257 | 0.824 | 1.01 (0.89-1.16) | 1.05 (0.92-1.20) | 0.98 (0.86-1.13) | 0.937 |
|  |  | Tanner 4 | 261 | 0.642 | 1.08 (0.95-1.22) | 1.08 (0.94-1.24) | 1.05 (0.91-1.20) | 0.532 |
|  | Percent Fibroglandular Volume | | | | | | | |
|  | CRP | Tanner 2 | 231 | 0.440 | 0.93 (0.84-1.03) | 0.95 (0.86-1.04) | 0.94 (0.85-1.04) | 0.237 |
|  |  | Tanner 4 | 263 | 0.793 | 0.98 (0.90-1.07) | 0.96 (0.89-1.04) | 1.00 (0.92-1.07) | 0.707 |
|  | IL-6 | Tanner 2 | 257 | 0.149 | 0.96 (0.88-1.06) | 0.93 (0.85-1.03) | 1.05 (0.94-1.16) | 0.441 |
|  |  | Tanner 4 | 261 | 0.219 | 0.95 (0.87-1.02) | 0.92 (0.85-1.00)* | 0.94 (0.86-1.02) | 0.119 |
|  | TNFR2 | Tanner 2 | 257 | 0.477 | 1.00 (0.91-1.10) | 0.97 (0.88-1.07) | 0.93 (0.84-1.03) | 0.142 |
|  |  | Tanner 4 | 261 | 0.407 | 0.94 (0.87-1.02) | 0.95 (0.88-1.04) | 0.98 (0.91-1.07) | 0.808 |
| **Multivariable-Adjusted Model^C^** | | | | | | | | |
|  | Total Breast Volume | | | | | | | |
|  | CRP | Tanner 2 | 223 | 0.486 | 1.10 (0.96-1.26) | 1.06 (0.93-1.21) | 1.07 (0.95-1.22) | 0.311 |
|  |  | Tanner 4 | 253 | 0.530 | 1.02 (0.91-1.15) | 1.01 (0.91-1.12) | 0.94 (0.85-1.05) | 0.367 |
|  | IL-6 | Tanner 2 | 249 | 0.809 | 1.02 (0.91-1.16) | 1.02 (0.90-1.16) | 0.96 (0.84-1.10) | 0.560 |
|  |  | Tanner 4 | 251 | 0.182 | 1.11 (1.00-1.24) | 0.99 (0.88-1.11) | 1.04 (0.92-1.16) | 0.950 |
|  | TNFR2 | Tanner 2 | 249 | 0.304 | 1.01 (0.90-1.14) | 1.11 (0.98-1.26) | 1.08 (0.95-1.23) | 0.131 |
|  |  | Tanner 4 | 251 | 0.089 | 1.13 (1.01-1.26)* | 1.13 (1.00-1.27)* | 1.04 (0.93-1.17) | 0.590 |
|  | Fibroglandular Volume | | | | | | | |
|  | CRP | Tanner 2 | 223 | 0.949 | 1.04 (0.90-1.20) | 1.02 (0.89-1.18) | 1.02 (0.89-1.18) | 0.756 |
|  |  | Tanner 4 | 253 | 0.821 | 0.99 (0.86-1.14) | 0.97 (0.86-1.10) | 0.94 (0.83-1.07) | 0.353 |
|  | IL-6 | Tanner 2 | 249 | 0.817 | 0.99 (0.87-1.13) | 0.95 (0.83-1.09) | 1.01 (0.87-1.17) | 0.957 |
|  |  | Tanner 4 | 251 | 0.260 | 1.05 (0.93-1.20) | 0.91 (0.80-1.05) | 0.97 (0.85-1.12) | 0.371 |
|  | TNFR2 | Tanner 2 | 249 | 0.690 | 1.02 (0.89-1.16) | 1.08 (0.94-1.24) | 1.01 (0.87-1.16) | 0.758 |
|  |  | Tanner 4 | 251 | 0.763 | 1.05 (0.92-1.20) | 1.07 (0.93-1.23) | 1.01 (0.88-1.16) | 0.816 |
|  | Percent Fibroglandular Volume | | | | | | | |
|  | CRP | Tanner 2 | 223 | 0.700 | 0.95 (0.85-1.05) | 0.97 (0.87-1.07) | 0.96 (0.86-1.06) | 0.405 |
|  |  | Tanner 4 | 253 | 0.733 | 0.98 (0.89-1.07) | 0.96 (0.89-1.04) | 1.00 (0.92-1.08) | 0.750 |
|  | IL-6 | Tanner 2 | 249 | 0.110 | 0.96 (0.87-1.06) | 0.93 (0.84-1.03) | 1.05 (0.95-1.17) | 0.380 |
|  |  | Tanner 4 | 251 | 0.268 | 0.95 (0.87-1.03) | 0.92 (0.85-1.00) | 0.94 (0.86-1.02) | 0.127 |
|  | TNFR2 | Tanner 2 | 249 | 0.449 | 1.01 (0.92-1.12) | 0.97 (0.88-1.08) | 0.93 (0.84-1.03) | 0.154 |
|  |  | Tanner 4 | 251 | 0.380 | 0.93 (0.86-1.01) | 0.95 (0.87-1.04) | 0.97 (0.89-1.06) | 0.710 |

^A^Linear regression model adjusting for age at inflammatory biomarker measurement

^B^Model adjusting for age at inflammatory biomarker measurement and fat percentage at biomarker measurement

^C^Model adjusting for age at inflammatory biomarker measurement, fat percentage at biomarker measurement, ethnicity, birth weight, height age- and sex-specific Z-score, and maternal education

^D^Likelihood ratio test (LRT) p-value for whether the addition of inflammatory biomarker quartiles improved model fit relative to the model without indicators for inflammatory biomarker quartiles

^E^Wald test p-value for log-transformed median within each quartile included as a continuous covariate in models adjusting for age at inflammatory biomarker measurement, fat percentage at biomarker measurement, ethnicity, birth weight, height age- and sex-specific Z-score, and maternal education

^F^Inflammatory marker quartiles at Tanner 2: CRP (mg/L): [0.1-0.3], [0.4-0.7], [0.8-2.2], [2.3-15.7]; IL-6 (pg/mL): [0.3-0.8], [0.9-1.3], [1.4-2.2], [2.3-30.4]; TNFR2 (pg/mL): [944.4-1894.1], [1897.2-2169.0], [2171.9-2533.7], [2545.9-4902.5]. Inflammatory marker quartiles at Tanner 4: CRP (mg/L): [0.1-0.2], [0.2-0.5], [0.5-1.9], [2.0-18.9]; IL-6 (pg/mL): [0.3-0.8], [0.9-1.3], [1.4-2.1], [2.2-24.0]; THFR2 (pg/mL): [1106.7-1960.4], [1971.3-2271.1], [2275.8-2638.1], [2639.9-4753.3].

* p <0.05

** p <0.01

***p<0.001
