# Supplementary material for: Association between indicators of systemic inflammation biomarkers during puberty with breast density and onset of menarche
Source: Breast Cancer Res. 2020 Oct 1;22:104. doi: 10.1186/s13058-020-01338-y (PMC7531086; doi:10.1186/s13058-020-01338-y)
Supplement: Supplementary file 5 — Additional file 5. Association of inflammatory marker measurement at Tanner 2 and Tanner 4 with relative time to menarche; restricting to individuals for which inflammatory markers were measured at Tanner 2 and Tanner 4. [file 13058_2020_1338_MOESM5_ESM.docx]

**Additional File 5. Association of inflammatory marker measurement at Tanner 2 and Tanner 4 with relative time to menarche; restricting to individuals for which inflammatory markers were measured at Tanner 2 and Tanner 4**

| Inflammatory Marker | Breast Tanner Stage | N | Events | Relative Time to Menarche Per Doubling of Inflammatory Marker; Time Ratio (95% CI) |
| --- | --- | --- | --- | --- |
| **Age-Adjusted Model^A^** | | | | |
| CRP | Tanner 2 | 238 | 220 | 1.00 (1.00-1.01) |
|  | Tanner 4 | 226 | 221 | 1.00 (1.00-1.01)** |
| IL-6 | Tanner 2 | 266 | 247 | 1.00 (0.99-1.00) |
|  | Tanner 4 | 225 | 220 | 1.00 (1.00-1.01) |
| TNFR2 | Tanner 2 | 266 | 247 | 0.98 (0.96-1.00) |
|  | Tanner 4 | 225 | 220 | 0.99 (0.97-1.01) |
| **Age and Body Fatness Adjusted Model^B^** | | | | |
| CRP | Tanner 2 | 237 | 219 | 1.00 (1.00-1.01) |
|  | Tanner 4 | 226 | 221 | 1.00 (1.00-1.01)** |
| IL-6 | Tanner 2 | 265 | 246 | 0.99 (0.98-1.00) |
|  | Tanner 4 | 225 | 220 | 1.00 (1.00-1.01) |
| TNFR2 | Tanner 2 | 265 | 246 | 0.98 (0.95-1.00)* |
|  | Tanner 4 | 225 | 220 | 0.99 (0.97-1.01) |
| **Multivariable-Adjusted Model^C^** | | | | |
| CRP | Tanner 2 | 229 | 211 | 1.00 (1.00-1.01) |
|  | Tanner 4 | 219 | 214 | 1.00 (1.00-1.01)** |
| IL-6 | Tanner 2 | 257 | 238 | 0.99 (0.98-1.00)* |
|  | Tanner 4 | 218 | 213 | 1.00 (1.00-1.01) |
| TNFR2 | Tanner 2 | 257 | 238 | 0.98 (0.96-1.00) |
|  | Tanner 4 | 218 | 213 | 1.00 (0.98-1.02) |

^A^Accelerated failure time model for time to menarche from birth adjusting for age at inflammatory biomarker measurement

^B^Model adjusting for age at inflammatory biomarker measurement and fat percentage at biomarker measurement

^C^Model adjusting for age at inflammatory biomarker measurement, fat percentage at biomarker measurement, ethnicity, birth weight, height age- and sex-specific Z-score, and maternal education

* p <0.05

** p <0.01

***p<0.001
