# Supplementary material for: Association between indicators of systemic inflammation biomarkers during puberty with breast density and onset of menarche
Source: Breast Cancer Res. 2020 Oct 1;22:104. doi: 10.1186/s13058-020-01338-y (PMC7531086; doi:10.1186/s13058-020-01338-y)
Supplement: Supplementary file 6 — Additional file 6. Association of quartiles of inflammatory markers measured at Tanner 2 and Tanner 4 with relative time to menarche; restricting to individuals for which inflammatory markers were measured at Tanner 2 and Tanner 4. [file 13058_2020_1338_MOESM6_ESM.docx]

**Additional File 6. Association of quartiles of inflammatory markers measured at Tanner 2 and Tanner 4 with relative time to menarche; restricting to individuals for which inflammatory markers were measured at Tanner 2 and Tanner 4**

|  | Inflammatory Marker | Breast Tanner Stage |  | |  |  | Relative Time to Menarche compared to Q1;  Time Ratio (95% CI)^F^ | | |  |
| --- | --- | --- | --- | --- | --- | --- | --- | --- | --- | --- |
|  |  |  | N | | Events | LRT^D^ | Q2 | Q3 | Q4 | Trend^F^ |
| **Age-Adjusted Model^A^** | | | | | | | | | | |
|  | CRP | Tanner 2 | 238 | 220 | | 0.215 | 1.00 (0.98-1.02) | 1.02 (1.00-1.04) | 1.00 (0.98-1.02) | 0.593 |
|  |  | Tanner 4 | 226 | 221 | | 0.003 | 0.98 (0.96-1.00)* | 1.00 (0.99-1.02) | 1.02 (1.00-1.03)* | 0.024 |
|  | IL-6 | Tanner 2 | 266 | 247 | | 0.588 | 1.01 (0.99-1.04) | 1.00 (0.98-1.02) | 1.00 (0.98-1.02) | 0.801 |
|  |  | Tanner 4 | 225 | 220 | | 0.450 | 1.00 (0.99-1.02) | 1.01 (1.00-1.03) | 1.01 (0.99-1.03) | 0.220 |
|  | TNFR2 | Tanner 2 | 266 | 247 | | 0.306 | 0.99 (0.97-1.02) | 0.99 (0.97-1.01) | 0.98 (0.96-1.00) | 0.064 |
|  |  | Tanner 4 | 225 | 220 | | 0.019 | 1.01 (1.00-1.03) | 1.01 (0.99-1.03) | 0.99 (0.97-1.01) | 0.174 |
| **Age and Body Fatness-Adjusted Model^B^** | | | | | | | | | | |
|  | CRP | Tanner 2 | 237 | | 219 | 0.197 | 1.00 (0.98-1.02) | 1.02 (1.00-1.04) | 1.00 (0.98-1.02) | 0.464 |
|  |  | Tanner 4 | 226 | | 221 | 0.004 | 0.98 (0.96-1.00)* | 1.00 (0.99-1.02) | 1.02 (1.00-1.03)* | 0.028 |
|  | IL-6 | Tanner 2 | 265 | | 246 | 0.519 | 1.01 (0.99-1.03) | 1.00 (0.97-1.02) | 1.00 (0.97-1.02) | 0.534 |
|  |  | Tanner 4 | 225 | | 220 | 0.495 | 1.00 (0.99-1.02) | 1.01 (0.99-1.03) | 1.01 (0.99-1.03) | 0.264 |
|  | TNFR2 | Tanner 2 | 265 | | 246 | 0.203 | 0.99 (0.97-1.02) | 0.99 (0.97-1.01) | 0.98 (0.95-1.00)* | 0.035 |
|  |  | Tanner 4 | 225 | | 220 | 0.018 | 1.01 (1.00-1.03) | 1.01 (0.99-1.03) | 0.99 (0.97-1.01) | 0.139 |
| **Multivariable-Adjusted Model^C^** | | | | | | | | | | |
|  | CRP | Tanner 2 | 229 | | 211 | 0.163 | 1.00 (0.98-1.02) | 1.02 (1.00-1.04)* | 1.00 (0.98-1.02) | 0.389 |
|  |  | Tanner 4 | 219 | | 214 | 0.003 | 0.98 (0.96-1.00)* | 1.01 (0.99-1.02) | 1.02 (1.00-1.03) | 0.023 |
|  | IL-6 | Tanner 2 | 257 | | 238 | 0.136 | 1.01 (0.99-1.03) | 1.00 (0.98-1.03) | 0.98 (0.96-1.00) | 0.089 |
|  |  | Tanner 4 | 218 | | 213 | 0.919 | 1.00 (0.99-1.02) | 1.01 (0.99-1.02) | 1.01 (0.99-1.02) | 0.534 |
|  | TNFR2 | Tanner 2 | 257 | | 238 | 0.098 | 0.99 (0.97-1.02) | 0.98 (0.96-1.00)* | 0.98 (0.96-1.00)* | 0.020 |
|  |  | Tanner 4 | 218 | | 213 | 0.041 | 1.02 (1.00-1.04)* | 1.02 (1.00-1.04) | 1.00 (0.98-1.02) | 0.732 |

^A^Accelerated failure time model for time to menarche from birth adjusting for age at inflammatory biomarker measurement

^B^Model adjusting for age at inflammatory biomarker measurement and fat percentage at biomarker measurement

^C^Model adjusting for age at inflammatory biomarker measurement, fat percentage at biomarker measurement, ethnicity, birth weight, height age- and sex-specific Z-score, and maternal education

^D^Likelihood ratio test (LRT) p-value for whether the addition of inflammatory biomarker quartiles improved model fit relative to the model without indicators for inflammatory biomarker quartiles

^E^Wald test p-value for log-transformed median within each quartile included as a continuous covariate in models adjusting for age at inflammatory biomarker measurement, fat percentage at biomarker measurement, ethnicity, birth weight, height age- and sex-specific Z-score, and maternal education

^F^Inflammatory marker quartiles at Tanner 2: CRP (mg/L): [0.1-0.3], [0.4-0.7], [0.8-2.2], [2.3-15.7]; IL-6 (pg/mL): [0.3-0.8], [0.9-1.3], [1.4-2.2], [2.3-30.4]; TNFR2 (pg/mL): [944.4-1894.1], [1897.2-2169.0], [2171.9-2533.7], [2545.9-4902.5]. Inflammatory marker quartiles at Tanner 4: CRP (mg/L): [0.1-0.2], [0.2-0.5], [0.5-1.9], [2.0-18.9]; IL-6 (pg/mL): [0.3-0.8], [0.9-1.3], [1.4-2.1], [2.2-24.0]; THFR2 (pg/mL): [1106.7-1960.4], [1971.3-2271.1], [2275.8-2638.1], [2639.9-4753.3].

* p <0.05

** p <0.01

***p<0.001
